# A Prospective Study of Spatial Clusters Gives Valuable Insights into Dengue Transmission

**DOI:** 10.1371/journal.pmed.0050220

**Published:** 2008-11-04

**Authors:** Steven Riley

## Abstract

Steven Riley discusses the public health implications of a new prospective spatial cluster study of dengue transmission in a rural population in Thailand.

If a school-age child contracts dengue in rural Thailand, it is difficult to predict whether that child was infected at home, at school, or somewhere else. Therefore it is difficult to know how best to allocate scarce resources towards public health measures to reduce the chance of further infections. However, the potential for transmission at home relative to the potential at school must depend mainly on two features of those settings: (1) the density of effective vectors compared with the human population, and (2) the previous infection history of the human population. Unfortunately, despite the wealth of knowledge that is available on many detailed aspects of the biology of dengue, firm ecological evidence with which to estimate key public health outcomes (such as the relative risk of transmission in different settings) remains surprisingly scarce. A prospective spatial cluster study of dengue transmission in rural Thailand by Mammen P. Mammen and colleagues reported in this issue of *PLoS Medicine* represents an important step forward in addressing this shortfall [1].

The global impact of dengue grew dramatically over the latter part of the 20th century, with the annual incidence currently estimated to be 50 million cases of dengue fever and 500,000 hospitalizations due to dengue hemorrhagic fever [2]. The pathogen is found throughout the tropics and subtropics (almost everywhere that viable vectors are found), with 2.5 billion people thought to be at risk [3]. No safe effective vaccine is currently available to prevent dengue infections. Therefore, public health strategies to reduce the adverse effects of the virus on human populations are designed (1) to reduce transmission by reducing vector abundance and (2) to mitigate morbidity using active surveillance and rapid supportive therapy [4]. Both strategies require substantial human resources that could be more effectively deployed if the patterns of dengue transmission at the local level were better understood. For example, although it is widely accepted that children are at an excess risk of infection with dengue, it is not clear if these extra infections occur at home, at school, or elsewhere.

**Linked Research Article**

This Perspective discusses the following new study published in *PLoS Medicine*:

Mammen MP Jr, Pimgate C, Koenraadt CJM, Rothman AL, Aldstadt J, et al. (2008) Spatial and temporal clustering of dengue virus transmission in Thai villages. PLoS Med 5(11): e205. doi:10.1371/journal.pmed.0050205

Investigating dengue cases identified by testing febrile schoolchildren in rural Thai villages, Mammen P. Mammen and colleagues find a pattern of focal spread to houses neighboring those of case patients.

## Children Were Infected Near Home

Mammen and colleagues used a prospective spatial cluster study to resolve some of these uncertainties for dengue transmission in a rural population in Thailand [1]. They started from a simple premise: during the time of year when dengue transmission is common, a substantial proportion of children who were absent from school because of illness (index cases) may have been infected recently with dengue. Therefore, absent children found to be infected with dengue (positive index cases) provided an ideal opportunity to directly observe possible spatial clusters of dengue infection in and around homes and schools. Children absent from school who were subsequently found not to be infected with dengue formed a perfect control group (negative index cases). The researchers recruited 33 index cases from 11 schools and 556 children from the households of index cases or nearby households (neighbors). Repeat blood samples were obtained (over 15 days of follow-up), and symptoms were recorded. The study team also tested adult mosquitoes in and around homes and schools, estimated numbers of Aedes aegypti pupae, and recorded key statistics of water usage.

All 27 dengue infections observed during follow-up in neighbor children were in positive clusters (initiated by a positive index case, *n* = 12). None of those 27 dengue infections were in negative clusters (*n* = 22). In other words, in this study, children who were absent from school because of dengue infection were always from very small geographical areas where dengue transmission was active, whereas those who were absent for other reasons were always from areas where dengue was not active. Therefore, these index cases were being infected by transmission near home. Also, of the 217 paired serological samples in the positive clusters, only one indicated dengue infection 60 days or more prior to enrollment, indicating that the positive clusters were probably due to a recent invasion. Data on mosquitoes near homes and schools supported the conclusion that children were infected near home.

## Prospective Cluster Studies

For the total resources used, the design of Mammen and colleagues' study was excellent. Their results give greater insight into the transmission dynamics of dengue than an earlier similar study in Indonesia (designed primarily to recruit early-stage infections for a pathogenesis investigation) [5]. For example, the researchers in Thailand recruited from schools rather than hospitals, included a control group, and conducted the study in a rural rather than an urban area (giving a greater chance of distinct spatial clusters) [1]. However, their study could certainly have been larger. As the authors note and discuss, their quantitative results are sensitive to the presence of one very heavily infected cluster and to the fact that one school was over-represented in the positive index cases. A high variance in the number of infections per cluster is an inherent characteristic of infectious disease transmission: for a given expected number of total cases, more clusters are required in infectious disease studies than would be required for chronic disease studies. With more clusters, the variations between clusters can be accounted for robustly when reporting key outcomes such as the underlying relationship between infection risk and distance.

In general, infectious disease epidemiology needs more prospective ecological studies. Unfortunately, many prospective cohort study designs are relatively expensive because of (1) the cost of recruiting so many individuals who will never be infected, and (2) the high staff costs of reactive follow-up by medical personnel. Without the immediately obvious public health benefits of an intervention, it is sometimes difficult to obtain funding for expensive ecological studies. Therefore, carefully designed prospective cluster studies provide a much more efficient way of gathering key data to improve our basic understanding of infectious disease transmission dynamics. Unlike in other branches of scientific investigation, even without explicit consideration of interventions, the wider societal benefits of improvements in our basic understanding of infectious disease dynamics are often rapidly apparent.
